# Integrating the Petrographic, Structural, Mechanical Characteristics, and Gamma-Ray Shielding Performance of Monzogranite as a Multifunctional Natural Material

**DOI:** 10.3390/ma19142935

**Published:** 2026-07-08

**Authors:** Mohamed Hasabelnaby, Mokhles K. Azer, Ghada Salaheldin, Ahmed E. Abdel Gawad, Saif M. Abo Khashaba, Mohamed Y. Hanfi

**Affiliations:** 1 Radiology and Medical Imaging Technology Department, School of Technology of Applied Health Sciences, Badr University in Cairo, Cairo 11829, Egypt; mohamed_hasab99@yahoo.com; 2 Department of Geological Sciences, National Research Centre, Cairo 12622, Egypt; mokhles72@yahoo.com; 3 Department of Physics, Faculty of Science, Assiut University, Assiut 71751, Egypt; ghadasalaheldin@aun.edu.eg; 4 Nuclear Materials Authority, El-Maadi, Cairo P.O. Box 530, Egypt; 5 Geology Department, Faculty of Science, Kafrelsheikh University, Kafrelsheikh 33516, Egypt; 6 Department of Life Safety, Institute of Fundamental Education, Ural Federal University, Ekaterinburg 620002, Russia; 7 Department of Physics, Dogus University, Dudullu-Ümraniye, 34775 Istanbul, Türkiye

**Keywords:** monzogranite, gamma-ray shielding, mechanical properties, petrographic characterization, natural shielding materials

## Abstract

This study describes a comparative assessment of the structural properties, mechanical properties and gamma-ray shielding effectiveness of monzogranite to determine whether or not they can be used for sustainable shielding construction materials. The results of the petrographic, X-ray fluorescence (XRF), X-ray diffraction (XRD), and energy dispersive spectroscopy (EDS) analyses reveal that the monzogranite is composed essentially of quartz, K-feldspar, plagioclase and biotite. The SiO_2_ contents of all the monzogranite studied also indicated that they are highly crystalline (70.77% to 73.34% SiO_2_ by weight) and chemically stable (therefore, monzogranite); other properties such as density (2.70 to 3.06 g/cm^3^), porosity (19 to 23%) and water absorption (12 to 15%) demonstrated the structural compactness and durability of the samples studied. Additionally, the mechanical properties of all of the samples were extremely high, and included: (a) the unconfined compressive strength ranged from 89.28 to 240.20 MPa; (b) the engineering modulus ranged from 40.6 to 66.5 GPa; (c) the Brazilian tensile strength ranged from 7.4 to 15.2 MPa; and (d) the flexural strength ranged from 9.3 to 16.4 MPa. The shielding effectiveness against gamma rays was rated over a wide range of photon energies (0.015–15 MeV) via Phy-X/PSD and experimentally using NaI (Tl) spectroscopy at specific gamma photon energies 0.662 MeV, 1.173 MeV and 1.332 MeV. The experimental measurements of gamma-ray attenuation were validated with Phy-X/PSD calculations, with the average variation being 5.8% and no single variation over 10%, and therefore, reliability has been successfully demonstrated. The linear attenuation coefficients (LACs) were measured from 24.674 cm^−1^ at 0.015 MeV to 0.065 cm^−1^ at 15 MeV, which illustrates the dependence of gamma-ray interactions’ mechanisms on the energy of the incoming radiation. The half value layer (HVL) went from 0.028 cm to 10.621 cm and the mean free path (MFP) increased from 0.041 cm to 15.323 cm. The best measured performance properties were attributed to specimen MB3, as it had the highest radiation protective efficiency (88.58% at 0.15 MeV) and the lowest radiation transmission (72.16% at 0.09 MeV) in comparison to all of the experimental conditions considered. The high attenuation properties of MB3 were attributed to its high density and high levels of iron oxide, Fe_2_O_3_. The present work demonstrates that monzogranite, specifically sample MB3, provides excellent mechanical strength, as well as effective shielding from gamma radiation. Therefore, monzogranite, and particularly MB3, is a creative alternative for sustainable construction, as it provides materials that will be used for radiation shielding in nuclear, medical and industrial applications.

## 1. Introduction

The dangerous impacts of ionizing radiation on both humans and the environment have generated worldwide apprehension, largely because of their application in agriculture, scientific research, healthcare, and energy production. These harmful effects include an increase in cancer rates, miscarriages, and stillbirths, along with physical and psychological disorders in infants [1,2,3,4]. Therefore, it is vital that protection against ionizing radiation ensures sufficient safety for employees at an acceptable level, thereby facilitating safe working conditions in environments where ionizing radiation is present; the protection of these ionizing radiations is mainly based on optimization principles [5,6,7,8,9,10].

Among all forms of ionizing radiation produced by radioactive materials, gamma radiation has proven to be the most challenging to handle; this is primarily due to the enhanced penetrating power of gamma rays [11,12,13]. Therefore, effective shielding from gamma radiation can be achieved through the use of solid-state materials that exhibit a relatively high density and strong radiation-shielding capabilities [14,15,16,17,18].

Conventional materials used for shielding against gamma rays are lead and concrete. However, due to the environmental hazards associated with lead and the susceptibility of concrete to chemical reactions and degradation from prolonged radiation exposure, researchers are exploring alternative materials that can effectively shield against gamma radiation while also being environmentally sustainable and economically viable [19,20,21,22]. Thus, alternative materials, such as natural resources like rocks, can be utilized as shielding materials because they are cost-effective and environmentally friendly. This strategy not only minimizes costs but also enhances ecological integrity. Therefore, the implementation of these materials is beneficial for both financial and environmental reasons. Granite rocks exhibit promising potential due to their high density, thermal and chemical stability, environmental friendliness, and resistance to corrosion [23,24,25,26].

Granitoids are widely distributed in the Egyptian Eastern Desert and Sinai. They were distinguished by different geochemical signatures, variable ages and tectonic settings [27,28,29,30]. These rocks were intruded in the Neoproterozoic basement complex, constituting nearly half of the basement rocks, particularly in the Eastern Desert. Granitoids can be categorized into three groups: (i) syn-collision calc-alkaline granites, (ii) late- to post-collision calc-alkaline granites, and (iii) post-collision alkaline granites. The syn-collision granites are composed mainly of highly and strongly deformed varieties, encompasses trondhjemite, tonalite and granodiorite; the late- to post-collision granites are distinguished by weakly deformed to undeformed granodiorite and granites; while the post-collision granites consist mainly of undeformed alkaline to peralkaline granites. The Egyptian granites are mostly distinguished by their compactness, fantastic colors and remarkable wide variety of durable appearances. They have their own fantastic appetences as ancient heritage, ornamental stones, modern constructions including paving, flooring, statues, cladding and funeral monuments [31,32,33]. The Egyptian post-collision granites have a crucial role in the most advanced high-tech applications, which attract many researchers due to their economic and strategic significance. These granites are enriched in rare metals, particularly Zr, Nb, Ta, Hf, Li, Sn, B, Be, Th, U, Y and REEs, that are widely used in hybrid electric vehicles, renewable energy applications, automobile industry, metal alloys, electronics, wind turbines, catalysts and other modern industrial applications [34,35,36,37].

**Figure 1:**
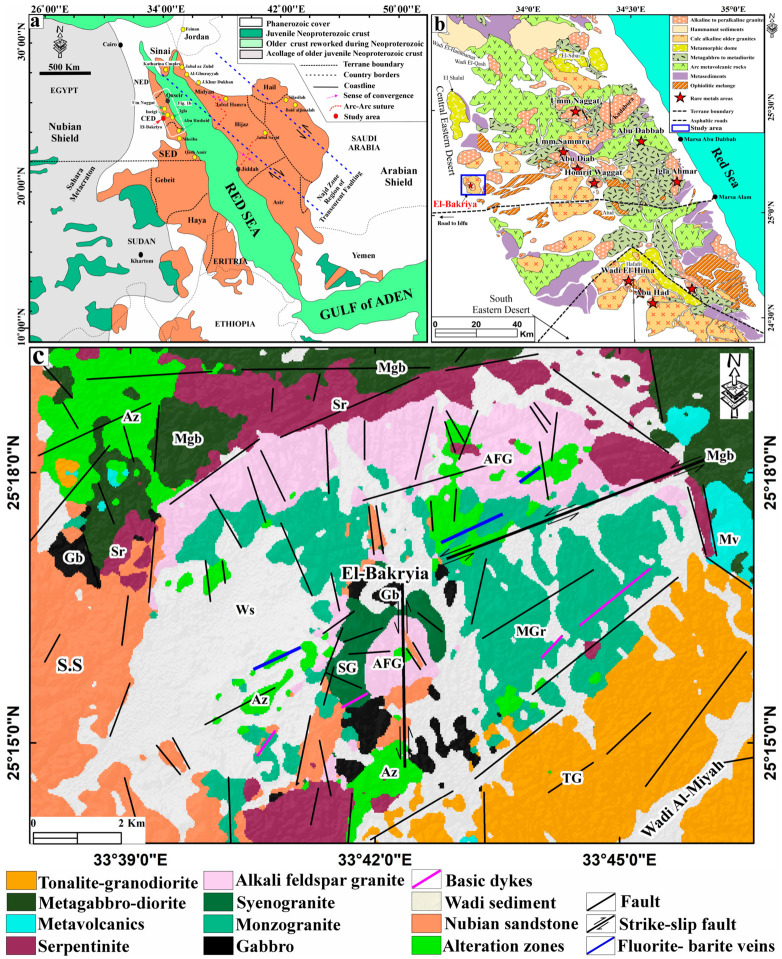
(**a**) Location map of the Arabian Nubian Shield (ANS) revealing the spatial distribution of some rare-metal alkaline granites and the investigated El-Bakriya ring complex, CED of Egypt [44]. (**b**) Geologic map of the Eastern Desert of Egypt, encountered the prospected El-Bakriya ring complex [45,46]. (**c**) Detailed geologic map of El-Bakriya ring complex, CED of Egypt modified after [39].

The prospected El-Bakriya region is well identified as one of the granitic rocks enriched in rare metals, which occurs in the Central Eastern Desert (CED) of Egypt as a part of the Arabian Nubian Shield (ANS) (Figure 1a,b). The El-Bakriya environs are of great interest to many researchers, coinciding with the uniqueness of granitic rocks bearing Zr, Nb, Ta, F, Ba, Ti, U, Th, and REE mineralization [38,39,40]. According to recent research, the engineering properties of granitic rock types are greatly influenced by factors such as the development of microcracks, thermo-mechanical deterioration, and the accumulation of damage from environmental conditions. Various experimental studies performed on granites demonstrate that repeated thermal cycling and cooling with liquid nitrogen lead to substantial changes in mechanical performance, fracturing morphology, and propagation of cracks in granites, which have an impact on the material’s strength and deformational behavior under load. Additionally, constitutive damage models using energy evolution techniques have been utilized successfully to estimate the degree of brittleness and failure mechanisms of geomaterials, providing valuable insight about crack initiation and propagation due to mechanical loading. These results highlight the need to consider the integration of mineralogical, physical, mechanical, and radiation-shielding properties in evaluating the overall engineering suitability of monzogranite for structural or shielding purposes [41,42,43]. The current study will investigate the potential of monzogranite as a multifunctional material suitable for construction and gamma-ray shielding through an integrated analysis of petrological properties, chemical composition, physical properties, mechanical properties and radiation attenuation characteristics. Through this research, we will determine the relationship between the minerals, density, porosity, and mechanical strength on the various parameters related to gamma-ray shielding such as linear attenuation coefficient, mass attenuation coefficient, half-value layer, mean free path, transmission factor, radiation protection effectiveness, effective atomic number, and effective electron density as a function of photon energy (0.015–15 MeV). The ultimate objective of this study is to determine the most effective varieties of the monzogranite for both sustainable structural and radiation protection applications (i.e., identify the most environmentally friendly shielding materials) and provide a scientific basis for using natural granitic materials as environmentally sustainable shielding materials.

## 2. Geologic Setting

**Figure 2:**
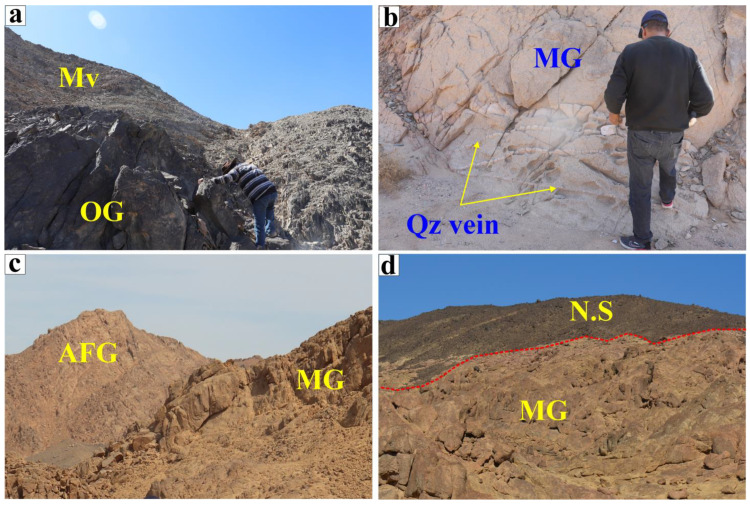
Field photographs of the monzogranite from El-Bakriya ring complex, CED, Egypt. (**a**) Sharp intrusive contact between older granitoids (OG) and metavolcanics (Mv). (**b**) Quartz (Qz) veinlets crosscut monzogranite (MG). (**c**) Sharp intrusive contact between monzogranite (MG) and alkali feldspar granite (AFG). (**d**) Nonconformity surface between the monzogranite (MG) and Nubian sandstone (N.S).

The El-Bakriya ring complex is situated in the southwestern part of the CED, and can be reached through the Idfu-Marsa Alam asphalt road and far from the El-Barramiya gold mine about 15 km to the north (Figure 1c). The investigated area reveals moderate relief except for the El-Bakriya plutons (~550 m above sea level), which show the highest peaks in the prospected region. The investigated El-Bakriya environs are mostly composed of the basement complex comprising older granitoids (tonalite-granodiorite, gabbro, younger granites (monzogranite, syenogranite, and alkali feldspar granite), and post-granite dikes, which were non-conformably overlain by Nubian sandstone of Cretaceous age (Figure 2a–d). The studied area displays a remarkable structural ring complex, and is mostly affected by different fractures, faults, and joints, as well as subsequent exfoliation, which have NE, NW, and N–S trends [39,40]. The older granitoids are mainly tonalite-granodiorite, and mostly occur as small scattered masses in the southeastern part of the prospected region. They show low-lying relief hills, with medium- to coarse grains, and whitish gray to grayish black colors, revealing remarkable exfoliation and bouldery appearances. These rocks enclose abundant xenoliths of metavolcanics from the surrounding country rocks, and show remarkable intrusive contact with such rocks (Figure 2a). These granitoids are intruded by younger granites with remarkable sharp intrusive contacts. Monzogranite appears as the most dominant younger granite in the environs investigated. It forms moderate to high relief hills, which are medium- to coarse-grained, with reddish pink to buff colors. It is characterized by hard, massive rock and is affected by different sets of faults system. Monzogranite is crosscut by quartz veinlets, and it displays a marked sharp intrusive contact with surrounding granitic plutons (Figure 2b,c). The mafic rocks are well represented by gabbro, which occurs as a massive, hard rock, with grayish green to black colors, as small, scattered outcrops in the northeast part of the prospected El-Bakriya environs. The gabbroic masses reveal low to moderate relief, mainly covered by alluvial deposits, and show a marked sharp intrusive contact with other rock types. The syenogranite appears as the outer rim of the ring complex, has a medium to coarse grain, with pinkish red to buff colors, and shows sharp contact with the non-conformable Nubian sandstone of Cretaceous age. Alkali feldspar granite appears as reddish pink to red and buff colors, medium- to coarse-grained, and its outcrops are characterized by the presence of quartz, fluorite and barite veins. The margin of the investigated alkali feldspar granite is highly sheared and is extensively altered. Sericitization, kaolinization, silicification and hematitization dominate hydrothermal alterations affecting the alkali feldspar granite, which results in an extensive brittle deformation (Figure 2c). The two conjugate fault systems NW and N–S are mostly predominant and are injected with quartz veinlets [39]. Pegmatite bodies are found along the periphery and outer margin of the investigated El-Bakriya alkali feldspar granite. Pegmatite occurs as a plug and/or is dike-like within the pluton and is distinguished by remarkable enrichments of rare metal mineralization [40]. Mafic dikes and/or pegmatite and aplite dikes as well as quartz and fluorite veins are observed traversing the granitic rocks. The Nubian sandstone non-conformably overlies the granitic rocks and gabbro as elongated outcrops (Figure 2d). The Nubian sandstone exposed in the core of the El-Bakriya ring complex and represents down faulted blocks that have NNE fault trends.

## 3. Materials and Methods

### 3.1. Chemical and Petrographical Analyses

For the microscopic examination, eleven representative thin sections of granitic samples have been prepared for petrographic analyses, with particular emphasis on mineralogical and textural features. All sample preparation has been carried out at the Department of Geology, Cairo University. These thin sections were studied by transmitted light under polarizing microscope (Nikon, Tokyo, Japan) equipped with a digital camera and many microphotographs were taken.

Based on the petrographic studies, the samples for different chemical analyses were selected. So, seven selected samples have been chosen from the monzogranite to determine the major oxides using ThermoARL XRF spectrometer (Thermo Fisher Scientific, Ecublens, Switzerland) as mentioned in Table 1. The chemical analyses have been done at the GeoAnalytical Laboratory, Washington State University, USA and the analysis of data was performed by [47]. The examined monzogranite samples have been crushed, powdered and ground by using agate to produce homogenous size fragments. The powdered monzogranite samples have been pulverized to ~40 mesh; each sample has subsequently mixed with two parts of di-lithium tetraborate flux, then fused at 1000 °C in a muffle furnace and cooled prior to be more suitable for chemical analysis. The resulting product is ground again, then re-fused, and followed by polishing on a diamond lap in order to produce flat and smooth surface. The GSP2 standards [48] from the USGS have been performed to be more suitable for calibration processes. The limits of the detection that have been determined for the major oxides are 0.01, as mentioned in Table 1, except Fe_2_O_3_, which reaches 0.04. Loss on ignition (LOI) has determined from the difference in weight for the same sample before and after heating at 1000 °C.

### 3.2. Preparation of Monzogranite Samples

**Figure 3:**
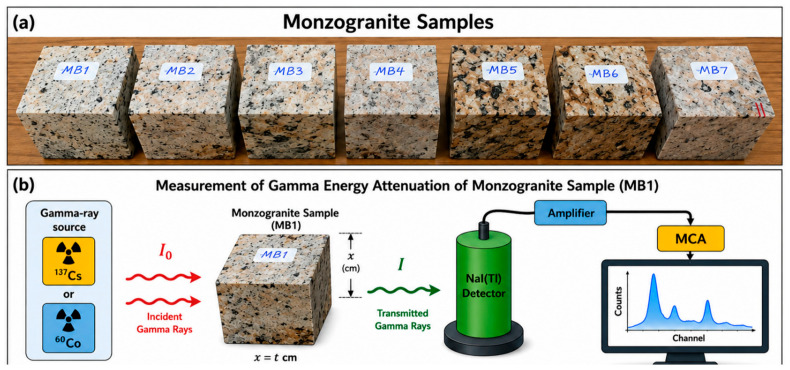
(**a**) Photograph shows the examined cubes of monzogranite from El-Bakriya ring complex, CED, Egypt. (**b**) The experimental setup for measuring the attenuation factors for monzogranite samples.

Seven representative monzogranite samples as shown in Figure 3 were cut to the specified dimensions 5 × 5 × 5 cm^3^ for the appropriate types of physical, mechanical, and radiological measurements to obtain shielding data. All specimens tested for gamma-ray attenuation had approximately equal thicknesses of 5 cm to provide mechanical stability and adequately attenuate radiation for the accurate determination of shielding parameters over the energy range of photon emissions tested. Through using appropriate counting times for all tests to provide acceptable results with adequate counting statistics, all recorded spectra had been corrected by removing background radiation before being analyzed. The monzogranite sample tested has a massive homogenous bulk structure that does not exhibit any visible foliation or preferred orientation of major minerals. Each sample was prepared from newly mined monzogranite and therefore cuts were not orientated to provide a preferred cutting direction; thus, all samples are indicative of the bulk monzogranite material.

### 3.3. Structural Analysis

The powdered monzogranite sample has identified by using X-ray diffraction (XRD) investigation. The XRD technique has been used for the determination of the mineralogical composition of the investigated monzogranite by using a PHILIPS PW 3710/31 diffractometer. The picked essential mineral grains have determined using Environmental Scanning Electron Microscope (ESEM) EXL 130, which was mounted to Energy Dispersive Spectrometer (EDS) unit (model Philips XL 30 ESEM, FEI/Philips Electron Optics, Eindhoven, The Netherlands). The data analyses have performed at these conditions: the counting times vary from 60 to 120 s, accelerating voltages range 25–30 Kv and the beam diameters vary from 1 to 2 mm. The detection limits varied from 0.1 to 1 wt%. The precision is well below 1%, and the accuracy of the results ranged between 2 and 10% for elements with Z > 9 (F) and varied from 10 to 20% for light elements (O, C, B, N and F). The analyzed monzogranite samples have been carried out at Nuclear Materials Authority of Egypt EDS and XRD laboratories.

### 3.4. Physico-Mechanical Properties

The density of the monzogranite samples was evaluated by calculating the mass (m) divided by the volume (V), as shown in Equation (1).

(1) $$\rho =\frac{m}{V}$$

The water absorption (K, %) values of all fabricated monzogranite samples were obtained according to the procedure defined in ASTM standards C642 [49]. The samples were oven-dried (105 ± 5 °C) to achieve constant mass before being cooled to room temperature under drying conditions. The dried weight (W_dry_) of each sample was recorded. The samples were then fully saturated by being submerged in distilled water for 48 h (to allow complete saturation), and subsequently boiled in water for 5 h (to allow the release of trapped air from available pore space). After cooling to ambient temperature while still submerged, the saturated surface dry weight (W_sat_) of each specimen and their submerged weight (Mi) were recorded. The K values were determined using ASTM C642 and the measured weights were used to calculate K. All measurements of K were replicated a minimum of three times (n = 3) before that property’s average and standard deviation were reported.

(2) $$K,\;\%=\frac{W_{sat}-W_{dry}}{W_{dry}}\;\times \;100$$

### 3.5. Mechanical Meaasurements

The compression strength test was carried out at Badr University in Cairo, Egypt, utilizing the ELE ADR-Auto V2.0 Standard Compression Machine (ELE International, Milton Keynes, UK), which boasts a high-precision, automated compression testing system. This system employs closed-loop technology to maintain accurate load rates and provides real-time plotting of load in relation to time. It is available in capacities of 2000 kN and 3000 kN, specifically designed for the testing of cubes and cylinders in line with BS EN ISO 7500-1 [50] and ASTM E4 standards [51].

The uniaxial compressive strength of the monzogranite sample indicates the greatest axial compressive stress that the sample can withstand before failure, without any lateral confinement. The maximum unconfined compressive strength is an essential measure of the strength and mechanical capabilities of a granite sample. The pressure applied to the samples is determined using the following Equation (3).

(3) $$\mathrm{UCS}=\frac{P}{A}$$

where P represents the maximum load (or the load at which failure occurs) applied to the material (N), and A denotes the cross-sectional area of the material that endures the load (m^2^).

The Young’s modulus value (E, GPa) was established by measuring the slope of the linear elastic region on the stress–strain graph; the below mentioned equation helps to calculate E [52].

(4) $$E\;(GPa)=\frac{\Delta \sigma }{\Delta \varepsilon }$$

where Δσ is the increase in axial stress (MPa), and Δε refers to the increase in axial strain (mm/mm).

The Poisson’s ratio (ν) value was determined by measuring axial and lateral strains. The equation below is used for this purpose.

(5) $$\nu =-\frac{\varepsilon_{lateral}}{\varepsilon_{axial}}$$

where $\varepsilon_{lateral}$ is lateral strain (mm/mm), and $\varepsilon_{axial}$ is axial strain (mm/mm).

Using the experimental values of E and ν above, the shear modulus (G), bulk modulus (B), and longitudinal modulus (L) were calculated using the following equations:

(6) $$G(GPa)=\frac{E}{2(1+\nu )}$$

(7) $$K(GPa)=\frac{E}{3(1-2\nu )}$$

(8) $$L=B+\frac{3}{4}\;S$$

The modulus ratio (MR) of the rocks was calculated using the formula:

(9) $$MR=\frac{E}{UCS}$$

The indirect tensile strength (BTS) of each rock sample was obtained using previous calculations and can be found using the following equation:

(10) $$BTS=\frac{2P}{\pi Dt}$$

where D is the diameter of the disc sample (mm) and t is the thickness of the sample (mm).

The flexural strength (MOR), or modulus of rupture, of each specimen was determined using the three-point loading apparatus as follows:

(11) $$MOR=\frac{3Pl}{2bd^{2}}$$

where l is the span length (mm), b is the sample width (mm), and d is the sample thickness (mm).

### 3.6. Gamma Spectrometric Analysis

Using the narrow-beam transmission technique, we performed gamma-ray transmission measurements to determine the radiation-shielding effectiveness of the monzogranite samples experimentally at the Nuclear Science Laboratory, Faculty of Science, Asyut University, Egypt. Our experimental setup included a 2″ × 2″ NaI (Tl) scintillation detector that was connected to a preamplifier, linear amplifier and 8K multichannel analyzer (MCA) for spectrum acquisition and analysis. The energy resolution of the detector was 5.37% at 662 keV; therefore, we could accurately identify and count the amount of gamma-ray photon data collected. Energy calibration of the detector was completed prior to each measurement using standard sealed gamma-ray reference sources. Monoenergetic gamma photons were produced by standard radioactive sources of ^137^Cs (662 keV) and ^60^Co (1173 and 1332 keV). These two gamma-ray sources were chosen because they provide a representation of the majority of the measured shielding materials in this investigation. Two lead (Pb) collimators with 10 mm apertures were placed between the source, sample and detector so as to produce a well-collimated narrow photon beam that would reduce or eliminate the effect of scattered radiation from reaching the detector. To maintain constant narrow-beam geometry for this whole experiment, the source, sample, and detector were carefully aligned in the same plane horizontally. The separation distance between the source and detector was set to a fixed value of 8 cm, and the sample of monzogranite was positioned equidistantly between the source and detector. The detector’s housing also contained extra lead shielding to reduce the effects of background radiation and to allow for more precise measurements of the number of photons counted. For each measurement, the photon spectrum was measured first without the sample, producing the measured incident (I_0_) intensity, then again with the sample inserted into the radiation beam path producing the transmitted (I) intensity. The measured background radiation was subtracted separately from both spectra prior to doing any analysis. A sufficient amount of time was allowed for the spectrum of each measurement to accumulate enough counts so that good counting statistics would exist and that statistical uncertainty could be minimized.

The reduction in radiation intensity as it moves through a target material is defined by parameter known as the linear attenuation coefficient (LAC); this coefficient is based on the density and atomic number of the material that absorbs the radiation. The LAC was calculated using the exponential formulation of Beier Lambert’s law, as indicated in Equation (12) [53,54,55].

(12) $$I=I_{0\;}e^{-LAC\;x}$$

and

(13) $$LAC\left(cm^{-1}\right)=\frac{1}{x}\ln\left(\frac{I_{o}}{I_{t}}\right)$$

where x is the thickness of the material, while I and I_0_ represent the counts captured by the detector after background subtraction, both with and without the material located between the detector and the source, respectively.

To find the uncertainty in LAC, we use error propagation. Assuming that I_0_, I, and x have uncertainties ΔI_0_, ΔI, and Δx, respectively, the uncertainty in μ can be expressed as:

(14) $$\Delta LAC=\sqrt{{{\left(\frac{{∆I}_{0}}{xI_{0}}\right)}^{2}+\;(\frac{∆I}{xI})}^{2}+{\left(\frac{\ln\left(\frac{I_{0}}{I}\right)∆x}{x^{2}}\right)}^{2}}$$

To eliminate the density effect, the mass attenuation coefficient (MAC, cm^2^/g) is introduced, obtained by isolating LAC by the experimental density (ρ) [56]:

(15) $$MAC=\frac{1}{\rho x}\ln\left(\frac{I_{0}}{I_{t}}\right)$$

Experimental values of the MAC obtained were compared to theoretical predictions generated by Phy-X/PSD software package available at https://phy-x.net/PSD (accessed on 15 May 2026) [57]. Database system was used to validate accuracy of experimental procedures. The Phy-X/PSD program utilizes data of interactivity in the form of photons provided by the National Institute of Standards and Technology (NIST) XCOM Database, which includes a full summary source of theoretical values for many energy and material combinations.

The half value layer (HVL) refers to the thickness of the shielding material that lowers the radiation intensity to fifty percent of its original level. These ideas are represented in Equation (16) [58,59].

(16) $$HVL=\frac{ln2}{LAC}$$

The mean free path (MFP) is an important parameter for evaluating the effectiveness of a material in terms of its radiation-shielding capabilities across different gamma energy levels. It is defined as the mean distance that gamma radiation can travel before it interacts with the shielding material, as outlined in Equation (17) [60].

(17) $$\mathrm{MFP}=\frac{1}{LAC}$$

Transmission Factor (% TF) is defined as the ratio of transmitted intensity relative to the original or incident intensity [61].

(18) $$\mathrm{TF}\;(\%)=\frac{I}{I_{0}}\times 100$$

The term radiation-shielding efficiency (RPE %) indicates the percentage of radiation that has been effectively mitigated or prevented, in relation to the expected level of attenuation or obstruction that the material used in the shielding method can provide, as shown in Equation (19).

(19) $$\mathrm{RPE}\;(\%)=[1-\;\frac{I}{I_{0}}]\times 100$$

## 4. Results and Discussion

### 4.1. Petrography of Monzogranite

**Figure 4:**
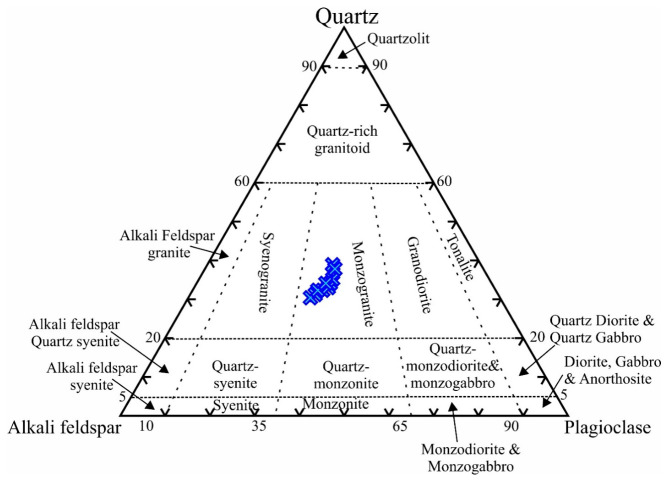
Ternary diagram shows the modal analysis (quartz–alkali feldspar–plagioclase) (QAP) of the investigated granite samples [62].

**Figure 5:**
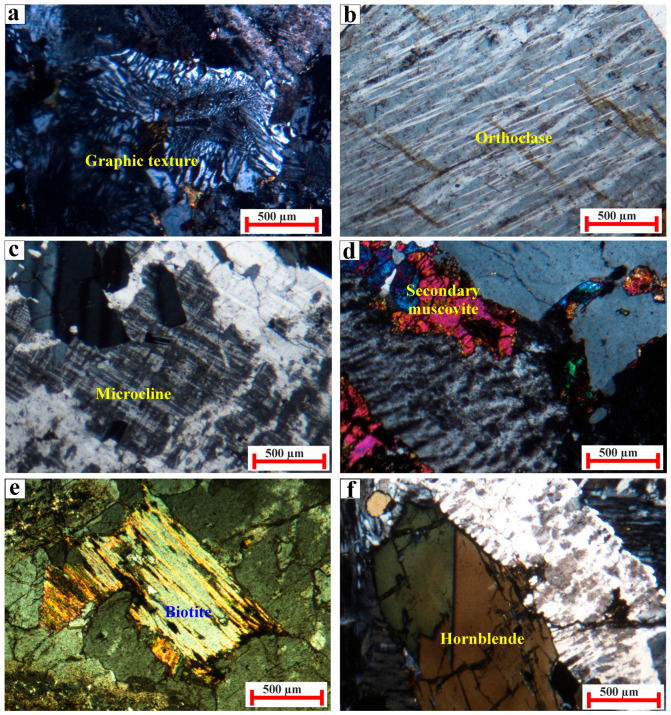
Photomicrographs of monzogranite in the Bakriya area. (**a**) Intergrowth of quartz and K-feldspar forming graphic texture. (**b**) Orthoclase containing albite lamellae forming ribbon perthite. (**c**) Microcline with crosshatch twining. (**d**) Fine aggregates of secondary muscovite formed at the expense of K-feldspars. (**e**) Highly altered and corroded biotite crystal. (**f**) Hornblende showing simple twining.

Monzogranite is characterized by pink to whitish buff and black colors and a coarse grain, and shows a hypidiomorphic granular texture. According to [62], the studied granitic samples are plotted in the field of monzogranite by utilizing the ternary diagram of the mineralogical modal classification (quartz-alkali feldspar-plagioclase) (QAP) as presented in Table 2 and Figure 4. The studied monzogranite consists mainly of K-feldspar, plagioclase, quartz, biotite and hornblende, with accessory phases comprising zircon, allanite, titanite, apatite and opaques, while the alteration products are sericite, chlorite, kaolinite and epidote (Figure 5a–f). K-feldspar is mostly dominated by microcline and orthoclase and shows remarkable intergrowth with quartz to form a micrographic texture (Figure 5a). Orthoclase appears as anhedral prismatic crystals, showing a marked simple twinning and compressed string, patchy, and flame perthitic intergrowths. It contains albite lamellae forming ribbon perthite (Figure 5b). Microcline forms subhedral prismatic to platy crystals, revealing a typical cross-hatched twinning (Figure 5c). Fine aggregates of secondary muscovite are formed at the expense of feldspars (Figure 5d). Plagioclase appears as subhedral prismatic crystals, some of them exhibit lamellar percline and simple twining; fine grains are poikilitically enclosed within the perthite. It is altered to sericite, particularly the inner core of crystals. Quartz occurs as anhedral to subhedral grains, filling the interstitial spaces between the other constituents. It displays undulose extinction, and some quartz crystals enclose microinclusions of microcline, albite and zircon. The mafic minerals are mostly dominated by biotite, with a few crystals of hornblende (Figure 5e,f). Biotite manifests as anhedral large flakes, which are highly corroded along their margins, and poikilitically enclose allanite inclusions. It is highly pleochroic, varied from pale brown to dark brown flakes, and showing abnormal interference colors (Figure 5e). It is engulfing some accessories such as apatite, zircon and opaques. It is highly altered to chlorite. Hornblende is rare, well presented as anhedral crystals that reveal simple twinning (Figure 5f). Zircon forms subhedral, prismatic crystals, which are colorless and display zonation. It is mostly enclosed in biotite, K-feldspar, quartz and hornblende. Allanite occurs as reddish-brown to burned brown colors, is zoned, and is often enclosed either in biotite or along the crystal’s rims. Titanite presents as a few euhedral sphenoid-shaped crystals, either encountered within biotite or K-feldspar. Apatite is found as a minute constituent as stalk-like or tabular crystals and is often enclosed in biotite. Opaque minerals occur as small grains, which are anhedral, and are usually disseminated in the altered feldspars, biotite and hornblende. They are mostly dominated by iron oxy-hydroxide materials.

### 4.2. Structural Characterizations

**Figure 6:**
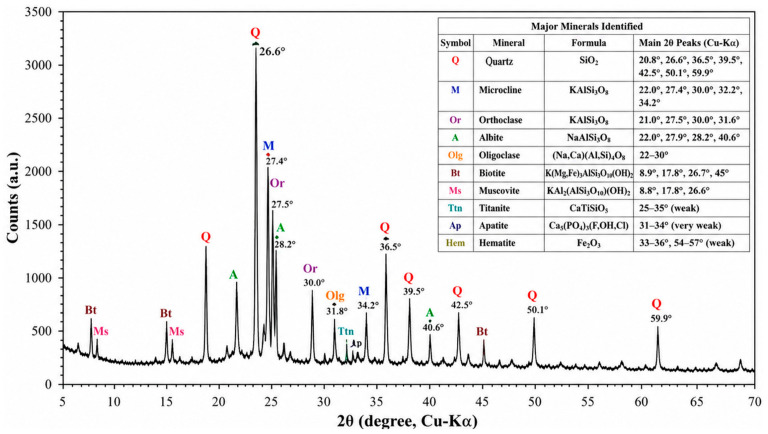
X-ray diffraction (XRD) patterns of the investigated monzogranite sample (MB1) prior to gamma irradiation.

The X-ray diffraction (XRD) patterns obtained from the analysis of the monzogranite samples (MB1 to MB7) prior to gamma irradiation show a characteristic high silica calc-alkaline granitic mineralogical composition (Figure 6). The shapes of the peaks observed on the diffractograms were relatively sharp, indicating that the mineralogical composition is well present. It is expected that the principal mineral phase in each of the samples studied will be quartz, which is substantiated by a marked peak located at approximately 26.6° (2θ), as well as several secondary peaks located at approximately 20.8°, 36.5°, 39.5°, 42.5°, 50.1°, and 59.9°. The dominance of quartz in the samples is also consistent with the high silica (SiO_2_) contents (70.77–73.34 wt%), as determined by XRF analyses. K-feldspars should be identified as the second most abundant mineral group in the monzogranite samples. Substantial diffraction peaks corresponding to microcline (KAlSi_3_O_8_) and orthoclase (KAlSi_3_O_8_) are expected between approximately 27° and 32° (2θ), consistent with the relatively high K_2_O content of (3.50–3.76 wt%). Additionally, sodium-rich plagioclase feldspar (albite, NaAlSi_3_O_8_) is also expected to produce visible peaks near approximately 22°, 28°, and 40° (2θ), which is consistent with the sodium oxide (Na_2_O) concentrations of (3.10–3.85 wt%). The co-occurrence of quartz and K-feldspar is indicative that in the examined monzogranite, the crystallization of the magmas occurred under silica saturation conditions. It is expected to find small diffraction peaks arising from biotite and muscovite in all sets as a result of the presence of some Fe_2_O_3_ (1.66–2.25%) and MgO (0.44–0.55%) in the materials. This is because of the low modal abundance of micas, which limit the amount of reflection they produce. Contained within the materials are small amounts of trace accessory mineralization, which include titanite (CaTiSiO_5_), apatite (Ca_5_(PO_4_)_3_(F,Cl,OH)) and iron oxides (hematite and/or magnetite), all of which may produce low-intensity peaks due to the small quantities of titanite, apatite and oxides of iron found in the samples, and are consistent with the measured TiO_2_ (0.11–0.31%), P_2_O_5_ (0.10–0.15%) and Fe_2_O_3_ contents. The small quantity of oxides of Ti, P, and Fe found in the samples indicates that accessory mineral assemblages represent only a minor percentage of the overall mineral assemblage of the samples. The distinctive and high intensities of the diffraction patterns indicate that the samples are highly crystalline and exhibit very little influence from amorphous material associated with dissolved solids. The secondary alteration minerals (chlorite, sericite and kaolinite) present in the monzogranite samples could be attributed to hydrothermal alteration. Evidence to support this conclusion includes the low loss on ignition (LOI) (0.56–1.10% LOI), which indicates small amounts of water and alteration processes affected the investigated monzogranite samples.

**Figure 7:**
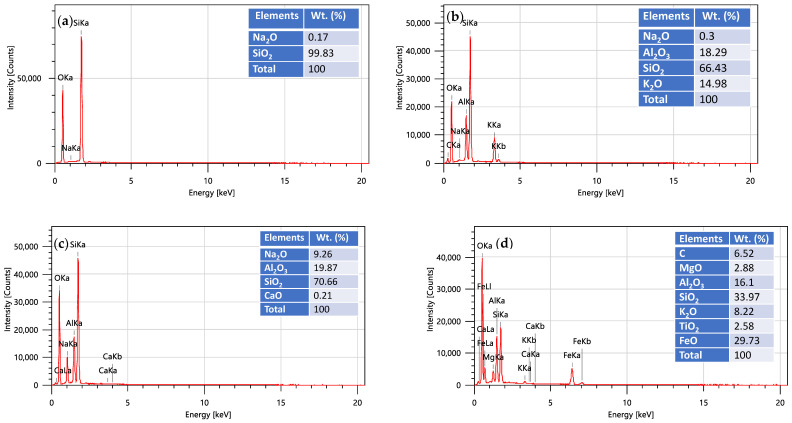
EDS analyses of the essential minerals including (**a**) quartz, (**b**) K-feldspar, (**c**) plagioclase and (**d**) biotite in the investigated monzogranite from El-Bakriya ring complex, CED, Egypt.

The findings from the EDS analyses in Figure 7a–d reveal that the mineralogical composition makeup of the monzogranite studied are consistent with the findings of the X-ray diffraction and X-ray fluorescence analyses. The major constituent mineral identified, quartz, was composed mainly of SiO_2_ (99.83 wt.%) confirming that quartz is a primary, pure-silica constituent phase of the monzogranite. The main components of K-feldspar are very high amounts of alumina (Al_2_O_3_) (18.29%), potassium oxide (K_2_O) (14.98%), and silica (66.43%), when referring to their mass percentage. In comparison, plagioclase (albite) contains high amounts of alumina (Al_2_O_3_) (19.87%), sodium oxide (Na_2_O) (9.26%), and silica (70.66%), based upon their mass percentage. Biotite, the other primary constituent mineral found at this aspect, possesses a significantly more variable mineral chemistry than that of K-feldspar or plagioclase; specifically, biotite contains approximately twice as much of the iron oxide (FeO) (29.73%) as its maximum, along with approximately 33.97% SiO_2_, 16.10% Al_2_O_3_, 8.22% K_2_O, 2.88% magnesium oxide (MgO) and 2.58% titanium oxide (TiO_2_), thus classifying biotite as an Fe-rich mica. There is a good correlation between the mineral chemistry derived from EDS analysis and the total rock chemical composition. Accordingly, the essential minerals of the monzogranite are quartz, K-feldspar, plagioclase, and biotite, and constitute the predominant rock forming minerals.

### 4.3. Physical Characterizations

**Figure 8:**
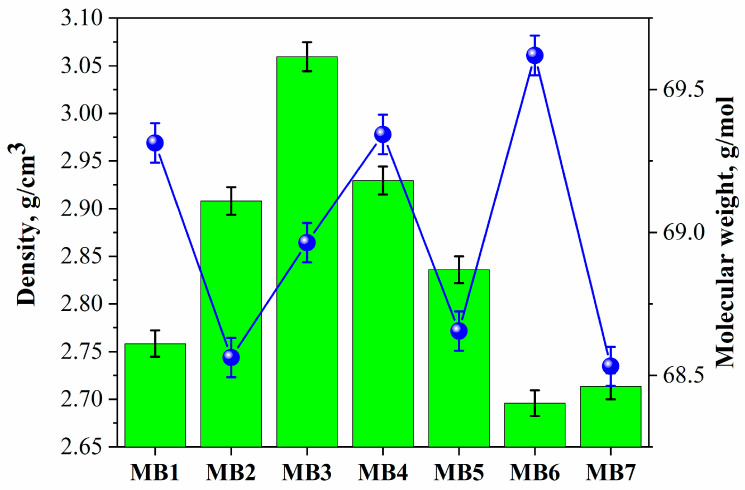
The density (g/cm^3^) in green color, and molecular weight (MW, g/mole) in blue color, of the natural monzogranite samples.

**Figure 9:**
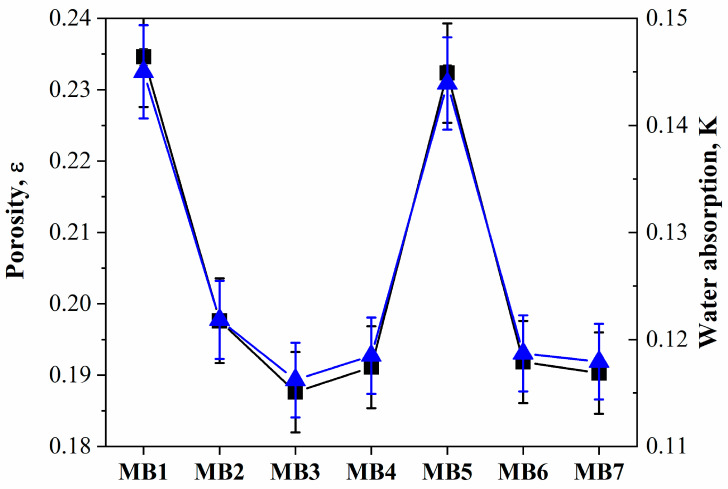
The porosity (ε) in black line and the water absorption coefficient (K, %) in blue line of the natural monzogranite samples.

Figure 8 and Figure 9 present the physical characteristics for the monzogranite samples examined (density, molecular weight, porosity, and water absorption). These characteristics significantly influence natural stone materials’ engineering behavior, durability and ability to shield against radiation. The density of the four monzogranite samples (MB3, MB4, MB2, MB6) in Figure 8 was tested and the densities ranged between 2.70 and 3.06 g/cm^3^. There were notable differences in density among the four specimens with MB3 being the densest at 3.06 g/cm^3^ and MB4 coming second at 2.93 g/cm^3^, then followed by MB2 at 2.91g/cm^3^. MB6 and MB7 had the lowest density (2.70 and 2.71 g/cm^3^). All of the measured densities fall in the typical density range for fresh monzogranite and show that there is variation in mineralogy and how the grains are packed among the different specimens. The relatively high density of specimen MB3 likely results from its relatively high concentration of primarily iron-bearing minerals, causing the sample to have a greater weight per unit of volume. The relatively lower densities of MB6 and MB7 likely result from their greater concentration of lighter silicate minerals and lower concentration of heavy minerals. The higher density of the material (MB3) will produce a greater probability of interacting with photons than the lower density material (MB6 and MB7), which explains the better performance of the higher density materials as radiation shielding, as stated previously.

Figure 8 indicates that the range of molecular weights, MW from 68.53 to 69.62 g/mole for the samples analyzed are all quite similar. MB6 (69.62 g/mole) and MB7 (68.53 g/mole) are two of the samples that were compared and they have similar molecular weights with only a small difference between them. The small difference in molecular weight demonstrates that the majority of monzogranite samples are chemically alike and consist predominantly of silica and aluminosilicate minerals. The differences in sample molecular weights are a result of variations in the relative amounts of significant oxides, such as SiO_2_, Al_2_O_3_, Fe_2_O_3_, CaO, Na_2_O, and K_2_O. Molecular weight is less directly related to the ability of a material to attenuate radiation than density is; however, it does play a role in establishing both the electron density and atomic packing characteristics of the rock matrix.

The measured porosity (ε) values for all samples in Figure 9 range between 19 and 23%, indicating very low pore volumes and a high density of internal structure for each sample. MB3, MB4, MB6, and MB7 showed the lowest values of porosity (19%), while MB1 and MB5 had the highest values of porosity (23%). Low porosity values are reflective of the crystalline and well-consolidated structural characteristics of monzogranite rocks, providing little or no interconnecting void spaces. Porosity is an important material characteristic that has a significant impact on both strength and the effectiveness as a shielding medium. A generally lower porosity value indicates denser and more compact materials, which yield greater amounts of atoms for every unit volume to interact with photons. Therefore, the low porosity samples (especially MB3 and MB4) should provide stronger attenuation characteristics than the more porous examples of MB1 and MB5 do. The consistent trend of increasing density as porosity decreases, with the densest sample, MB3, exhibiting one of the lowest values of porosity, provides additional evidence in support of this conclusion.

Water absorption results in Figure 9 ranged from 12% to 15%, indicating very low permeability and highly compacted samples. The lowest water absorption values (12%) were seen for MB2, MB3, MB4, MB6, and MB7; however, the highest values (14% and 15%) were for MB1 and MB5, respectively. The low capacity for absorption of water is associated with a poorly connected pore system and low porosity of the monzogranite samples. Fewer open pores allow for lower rates of moisture penetration, leading to a reduction in the total quantity of moisture absorbed. This quality would be highly desirable in construction and shielding applications due to the increased long-term durability of materials, reduced susceptibility of materials to weathering, and preservation of material structural integrity during varying environmental conditions. The correlation between porosity and water absorption is clearly positive; MB1 and MB5 have higher porosity values than other samples in this study, and thus exhibit a greater ability to absorb water. On the other hand, samples with lower porosities generally exhibit lower water absorbing capacities. Therefore, it is clear that the volume of the pore system and the connectivity of those pores is the primary factor influencing the moisture absorption capacity of the study’s monzogranite samples.

### 4.4. Mechanical Properties

The different mechanical behaviors of the monzogranite samples were quantitative as a result of the values for the following mechanical properties: compressive strength (UCS), Young’s modulus (E), Poisson’s ratio (v), Brazilian tensile strength (BTS), flexural strength (MOR), shear modulus (G), bulk modulus (K), longitudinal modulus (L), and modulus ratio (MR). The results of these tests are summarized in Table 3. The strength, stiffness and deformation characteristics of the monzogranite samples are all significantly different and are related to their mineralogical compositions and internal microstructural fabrics. In terms of UCS, the monzogranite exhibited a wide range of values, from 89.28 MPa (MB6) to 240.20 MPa (MB7), which means the monzogranite are moderate to very strong according to standard rock mass classification systems. The values for MB7 (240.20 MPa) and MB5 (224.86 MPa) are the highest values recorded and, therefore, demonstrate the densest micro-structure and the greatest degree of interlocking of the constituent grains. In comparison, MB6 and MB1 had much lower UCS values of 89.28 MPa and 98.32 MPa, respectively, which is attributed to a relatively high density of micro-cracks and weaken mineral bonding within the fabric of the rock.

With Young’s Modulus (E) ranging from 40.6 ± 1.1 GPa to 66.5 ± 1.8 GPa and a strong positive correlation with UCS, both MB7 and MB5 have the highest E values (66.5 GPa and 63.8 GPa, respectively) due to their more rigid internal structure resulting in less elastic deformation underload. Conversely, MB6 shows the lowest E value (40.6 GPa) indicating that it is the weakest in terms of mechanical structure. Poisson’s ratio is consistently between 0.21 and 0.25 across the samples suggesting that the elastic lateral deformation is consistent for all of the samples. The slightly elevated values of MB5 and MB7 (ν = 0.25) indicate a slightly larger potential to develop lateral strain due to axial load. Brazilian tensile strength (BTS) is from 7.4 MPa to 15.2 MPa, while the MOR is from 9.3 MPa to 16.4 MPa with both MB6 and MB7 having the highest tensile and flexural strengths. Therefore, they exhibit the best tensile and bending resistance characteristics. In general, these results correspond to UCS trends where samples with higher compressive strengths also show better tensile and flexural performances due to greater cohesion among grains and less porosity. Shear modulus (G) is 16.8 GPa to 26.6 GPa and bulk modulus (K) is 23.5 GPa to 44.3 GPa; MB7 and MB5 have the highest values for both. This corresponds to MB7 and MB5 having a greater ability to resist deformation through shear or volumetric compression. Longitudinal modulus of elasticity (L) varies from 63.0 GPa to 102.0 GPa for MB6 and MB7 respectively, confirming once again the elastic rigidity of MB7. As a result, it can be concluded that MB7 and MB5 have the highest elastic deformation resistance characteristics regardless of the type of load (shear, volumetric, axial) making them the most mechanically appropriate choice for applications needing high stability and high load bearing capacity. The Modulus Ratio (MR), which is the ratio of the modulus of elasticity (Young’s Modulus) to unconfined compressive strength (UCS) for each of the various lithologies, ranges from 277 (MB7) to 455 (MB6). The higher modulus ratio of MB6 and MB1 indicates there is relatively less stiffness when compared to their compressive strength, signifying a rock matrix that is more deformable. Conversely, the lower modulus ratio of MB7 and MB5 represents a more balanced relationship between stiffness and strength of the material and is typically indicative of more competent and structurally stable rock materials. The mechanical performance of the materials shows a close association between compressive strength and both the elastic modulus of the material and how they respond to tension. The samples MB7, MB5, and MB2 consistently outperform all of the other materials in all types of mechanical parameters, resulting in high UCS, high elastic modulus values, and high tensile and flexural strengths. This is a result of having a denser packing of the minerals, a stronger interlocking of the quartz and feldspar, and fewer micro fractures within the materials. Conversely, samples MB6 and MB1 have consistently demonstrated lower mechanical performance and therefore indicate weaker internal connections between particles and higher potential for deformation/degradation. From an engineering perspective, materials MB7 and MB5 are the preferred choices for structural and radiation shielding due to their superior strength, stiffness, and complete mechanical stability, while MB6 is the least desirable of all the tested candidates.

### 4.5. Radiation-Shielding Features

**Figure 10:**
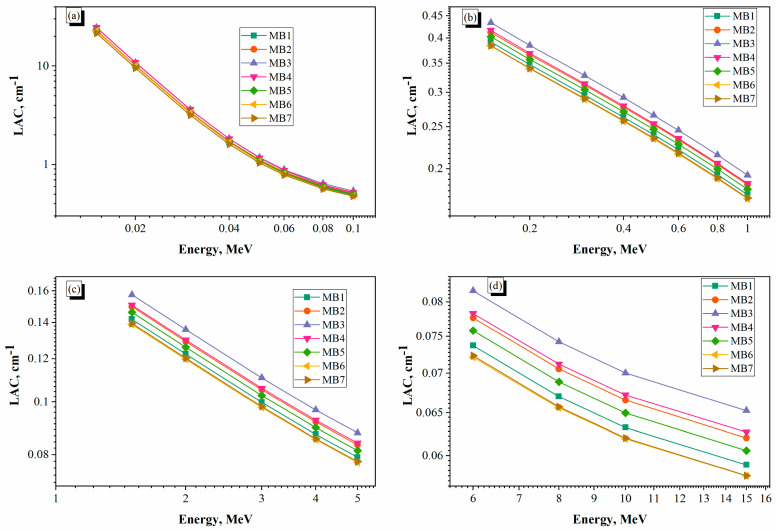
The influence of gamma-ray energy (MeV) (**a**) 0.015–0.1 MeV, (**b**) 0.15–1 MeV, (**c**) 2–5 MeV and (**d**) 6–15 of studied MB1, MB2, MB3, MB4, MB5, MB6 and MB7 natural monzogranite samples.

Figure 10a–d shows that the LAC will be used to determine the ability of different granitic rocks to attenuate gamma radiation from an MeV energy level of 0.045 to 15 MeV, while the coefficients of LAC will provide a direct indicator of the shielding characteristics of each granite type. Granitic sample analysis indicates that chemical composition analysis shows that all granite sample groups contain high amounts of SiO_2_ (70.77–73.10 wt%) and Al_2_O_3_ (14.76–15.62 wt%) and lower amounts of alkali metals, whereas granite samples contain Fe_2_O_3_ (1.68–2.25 wt%). Therefore, due to the various chemical compositions of the granite samples, the way in which each monzogranite sample interacts with gamma radiation will be greatly impacted and therefore LAC values will greatly affect how much gamma radiation can be attenuated by each granite sample. The LAC values obtained in Figure 10a exhibited a clear relationship with gamma photon energy level determining that the highest degree of attenuation of gamma rays was observed in the lower range of energies where the attenuation coefficient decreased from 22.740 to 24.674 cm^−1^ over a gamma-ray energy range of 0.015–0.1 MeV. The attenuation of gamma rays by sample MB4 was found to be 24.674 cm^−1^ at 0.015 MeV while sample MB7 had the lowest gamma attenuation of the 16 investigated samples at 21.623 cm^−1^. The rapid decline in LAC over this energy range is due largely to the fact that the dominant interaction is a photoelectric absorption process with a probability of interaction that varies significantly with energy and effective atomic number per the following relationship: σ_pe_ ∝ Z^4–4.5^/E^−3.5^, where σpe is the photoelectric cross-section, Z is the atomic number of the element and E is the photon energy [63,64,65,66,67,68]. The substantial decrease in LAC between 0.015 and 0.1 MeV indicates that, as expected, energy has an inverse relationship with the photoelectric cross-section. Samples MB3 and MB4 showed consistently better attenuation properties due their relatively high concentrations of Fe_2_O_3_, CaO and K_2_O, which increases the effective atomic number of the rock matrix. The Compton scattering interaction process is the dominant interaction in this intermediate energy range (0.1–1 MeV) as presented in Figure 10b. The ratio of the Compton scattering cross-section to the number of electrons in a material is approximately proportional to the number of electrons in the material. σ_CS_ ∝ Z/A, where A = atomic mass. Since the Z to A ratio varies very little from one element to another within the grains of monzogranite, the differences between LAC performance begin to become less significant than those seen in the photoelectric region [58,69,70,71]. However, MB3 continued to demonstrate the highest LAC across the whole of this energy range (reaching 0.193 cm^−1^ at 1 MeV), while MB6 demonstrated the lowest LAC (0.170 cm^−1^). As a result of the lesser influence of element composition on the sensitivity of Compton scattering to LAC, the difference in LAC between the different samples becomes progressively reduced with increasing energy.

As energy increases (>1.022 MeV) in the range 1–5 MeV in Figure 10c and in the range 6–15 MeV in Figure 10d, pair production plays a major role in the attenuation of photons. The LAC values decreased from 0.139 to 0.157 cm^−1^ at 1.5 MeV to 0.058–0.065 cm^−1^ at 15 MeV. The cross-section for pair production can be roughly calculated as: σ_pp_ ∝ Z^2^ln E. Thus, it has a quadratic relationship with Z and a logarithmic relationship with E [72,73,74,75]. Even though pair production becomes more important than Compton scattering over 1.022 MeV, the overall attenuation coefficients decrease due to more of a decrease in Compton scattering than an increase due to pair production. Therefore, all samples exhibit a gradual decline in LAC values with increasing energy. At 15 MeV, MB3 had the highest attenuation coefficient (0.065 cm^−1^) and MB6 and MB7 had the lowest (0.058 cm^−1^). This pattern corresponds with some degree of lateral influence on pair production via the increase in effective atomic number associated with the proportionally higher Fe_2_O_3_ content present in MB3.

The mineralogical and chemical composition of monzogranite is ultimately controls their efficiency at shielding. Samples MB3/MB4 also demonstrated very good rates of attenuation for all of the samples throughout the entire energy range of interest when detected. The primary reason for this was due to the higher concentrations of Fe_2_O_3_ (1.81–2.24%), CaO (1.23–1.88%) and K_2_O (3.53–3.74%) contained in these two samples, which lead to a higher effective atomic number and increased electron density. On the other hand, both samples MB6 and MB7 showed much poorer rates of attenuation; however, both have similar concentrations of SiO_2_. They also had lower concentrations of heavy oxide elements than other samples, which in return results in decreasing the probability of photoelectric absorption or pair production. Therefore, the two samples had lower average attenuation or ‘mean’ values. The trend of mean attenuation coefficients is: MB4 ≈ MB3 > MB2 > MB5 > MB1 > MB6 ≈ MB7. This trend was consistent for the entire energy range studied.

The comparison between the MAC values obtained from experimental data versus those produced from Phy-X/PSD simulations demonstrated that the two data sets demonstrate good agreement, with percentage differences ranging from approximately 3% to 8% over the energy range studied. Presented in Table 4 at 0.662 MeV, the percentage difference between the test and simulation data ranges from 3.52% (MB4) to 8.21% (MB3); whereas at 1.173 MeV this percentage difference lies within the range of 3.60% (MB4) to 8.40% (MB6). Discrepancy between experimental and simulated values for the 1.332 MeV energy level was also noted; however, this difference decreased slightly (3.47% (MB7) through 8.06% (MB4)). All sample experiments conducted have yielded MAC values slightly lower than the corresponding theoretical values obtained in this study, with an average deviation of approximately five percent (5.8%) between the experimental data and those predicted by the Phy-X/PSD software; hence, all MAC experimental data closely correlate with theoretical data. The various inconsistencies recorded can be attributed primarily to the natural variation of the monzogranite investigated. Natural rock has local variations in mineral composition, size of grains, and the distribution of heavy accessory minerals such as zircon, titanite, allanite and opaque materials. Although the theoretical calculations assume that the material is entirely homogeneous with uniformly distributed elemental constituents; this is not the case for natural rocks. The mineralogical, radiographic and structural inhomogeneities inherent to these types of rocks will affect both the effective path lengths and the scattering probabilities of photons; therefore, it is expected that there will be small discrepancies between the measured and calculated attenuation coefficients.

Additionally, experimental uncertainties related to density determinations, specimen thickness measurements, detector calibrations, photon counting statistics, and background corrections contribute to the discrepancies observed. Nonetheless, the deviations of all measurements from their theoretical counterparts remain less than 10%, thereby verifying that there is good agreement between the experimental measurements and theoretical calculations and providing confidence in both the experimental techniques used and the Phy-X/PSD computational methods.

**Figure 11:**
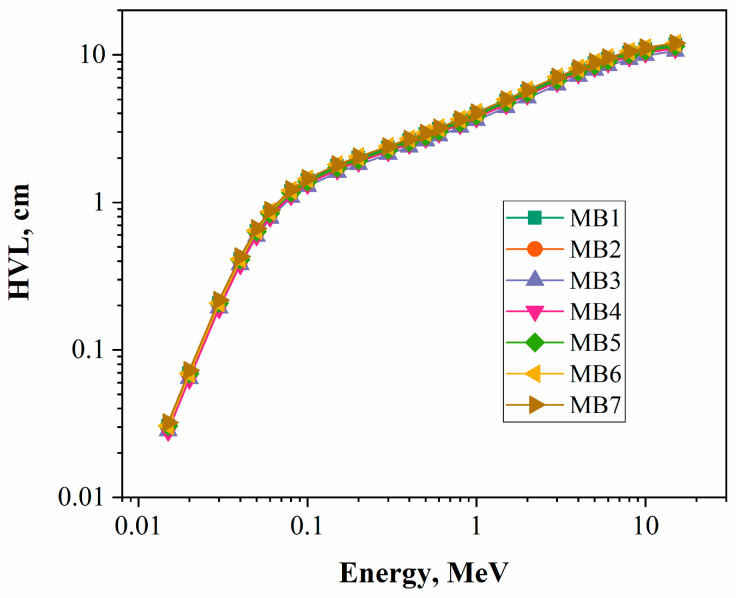
Variation of the HVL (cm) values at gamma energy range (0.015–15 MeV) of the studied monzogranite samples.

The half-value layer (HVL) values of the studied monzogranite samples in Figure 11 were considerably dependent upon photon energy and increased from the lowest energy (0.015 MeV) to the highest (15 MeV). Specifically, at an energy of 0.015 MeV, the HVL values ranged from 0.028 to 0.032 cm, whereas at 15 MeV the HVL values had increased to a range of 10.621 to 11.998 cm, which illustrates a need for thicker shielding material to attenuate high-energy photons than are required for low-energy photons. The continuous decline in linear attenuation coefficient (LAC) with increasing energy is directly related to this trend based upon an inverse relationship of HVL = ln(2)/LAC. The HVL values were very low at the lower photon energies, where photoelectric absorption dominates, and this is due to the high ability of the studied monzogranite to attenuate photons. At 0.015 MeV, for example, the HVL values of samples MB3 and MB4 were the lowest (0.028 cm), whereas sample MB7 had the highest HVL value (0.032 cm). The HVL values increased gradually as photon energy increased into the Compton scattering region (0.1 to 1 MeV) to a range of 3.586 to 4.066 cm at 1 MeV, due to the lesser probability of interaction between photon and matter. With regard to the area of high energy (>1 MeV), since both Compton scattering and pair production are factors for contributing to the HVL, and the HVL continued to increase as the energy increased, with the maximum HVL values reached at 15 MeV, the highest HVL was noted for the MB3 at all the energies studied, being noted as decreasing from 0.028 cm at 0.015 MeV to 10.621 cm for 15 MeV. MB4 and MB2 had similar results, but this can be primarily attributed to their greater densities and relatively greater amounts of heavier oxides in the material such as Fe_2_O_3_, which facilitated the attenuation of photons and reduced the thickness of material required for the attenuation of incident radiation by 50%. The MB6 and MB7 samples had HVLs that were typically the highest, being noted as 11.998 cm and 11.995 cm at the 15 MeV energy, respectively, indicating that these materials were less effective at attenuating radiation than the other samples that were evaluated. Overall, the samples were ranked in shielding performance based on HVLs as MB3 > MB4 > MB2 > MB5 > MB1 > MB7 ≈ MB6, which was in agreement with the trends for linear attenuation coefficients. The results of this study show that the monzogranite samples, especially MB3 and MB4, have good gamma-ray attenuation characteristics, and relatively thicker monzogranite materials are required for effective radiation shielding, which are therefore good candidates for use in protective construction and radiation-shielding applications.

**Figure 12:**
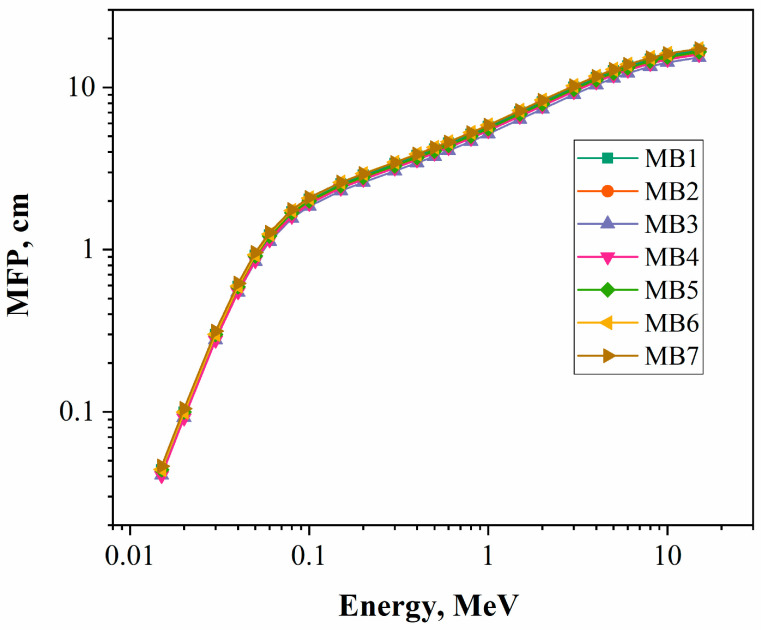
Variation of the MFP (cm) values at gamma energy range (0.015–15 MeV) of the studied monzogranite samples.

Figure 12 reveals that MFP values for the monzogranite samples increased systematically with increasing energy over the entire energy range observed. As the energy of the photon increases, the probability that a photon will interact with a rock matrix decreases and therefore the mean free path increases. At low energy levels (0.015 MeV), the mean free path is about 0.041–0.046 cm, while at high energy levels (15 MeV) the mean free path is about 15.323–17.309 cm. Since the mean free path is inversely proportional to the linear attenuation coefficient, this behavior is expected. As the linear attenuation coefficient decreases, so will the mean free path of the photon. Photons are therefore able to travel longer distances between the time they enter the sample and the time they interact with the material as the attenuation coefficients decrease. Among the samples investigated here, MB3 had the consistently lowest range of MFP values compared to the other samples for the entire investigated energy range from 0.041 at 0.015 MeV to 15.323 at 15 MeV, indicating that MB3 had the highest probability of photon interaction and the best shielding ability. Conversely, MB6 and MB7 had the highest MFP values for the displayed range, with MFP values of 17.309 cm and 17.306 cm, respectively, at 15 MeV, indicating that MB6 and MB7 had a lower efficiency for attenuating photon interaction probability. The relationships observed between MFP and density and chemical composition were related to the densities of the individual monzogranite samples that corresponded to the amount of heavier oxides in each sample, which would provide the incident photons with increased total interaction sites as opposed to those samples with lower densities.

**Figure 13:**
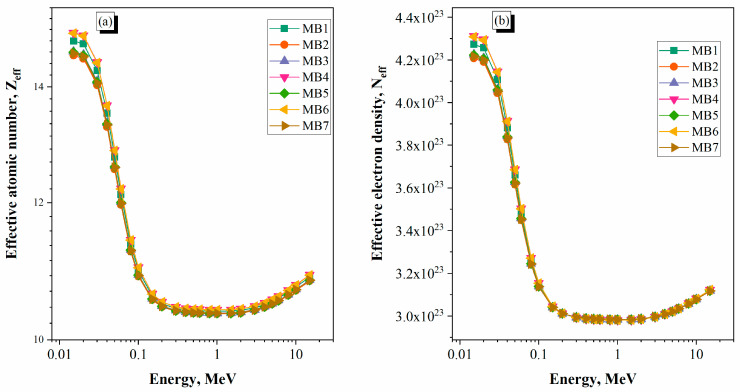
Variation of (**a**) the effective atomic number (Z_eff_) and (**b**) effective electron density (N_eff_) values at gamma photon energy range of (0.015–15 Mev) in the studied monzogranite samples.

An analysis of the effective atomic number (Z_eff_) of the monzogranite samples examined demonstrates a characteristic energy-dependent trend and is presented in Figure 13a: it initially decreased rapidly in the low energy region, then remained almost stable throughout mid-range energies, and then increased slightly at high energy ranges. At 0.015 MeV, Z_eff_ values were between 14.04 and 15.05, followed to approximately 10.35–10.43 during the Compton scattering region (0.3–1 MeV), and finally increased slightly to 10.81–10.90 at 15 MeV. The generally elevated Z_eff_ value at low energies results from the dominance of the photoelectric absorption process that heavily depends on atomic number (Z^4–4.5^); however, as the energy increases and the contribution of the Compton scattering process takes over, the influence of the atomic number becomes less, thus the near constant Z_eff_ value observed across the mid-range energies. An increase in the Z_eff_ value at higher energy levels is caused by the addition of pair production contributions, which has a cross-section proportional to Z^2^. Samples MB4 and MB6, which both had the largest Z_eff_ values (15.05 and 15.04 at 0.015 MeV, respectively), consistently outperformed sample MB2. Therefore, it can be inferred from this observation that the larger Z_eff_ values of both MB4 and MB6 (when compared with sample MB2) indicate an increased ability of MB4 and/or MB6 to not only interact with photons but also absorb more photons than any other samples in this test.

The effective electron density, N_eff_, values for the monzogranite samples in Figure 13b show a similar pattern to that of Z_eff_ and follow the different types of photon interactions within the different energy ranges investigated. N_eff_ decreased from about (4.05 × 10^23^–4.31 × 10^23^) electrons cm^−3^ at (0.015 MeV), down to about (2.98 × 10^23^–3.01 × 10^23^) electrons cm^−3^ through the intermediate energy range and then increased gradually to about (3.12 × 10^23^) electrons cm^−3^ as the energy reaches (15 MeV). The higher N_eff_ values at lower energies correlate to the photoelectric absorption region where electron interaction with photons is most important. As we enter the Compton scattering region, the electron density values level out because the probability of interaction occurs based on the number of electrons available for interaction, rather than the atomic number. As we reach the higher energy range, the slight increase in Neff results from the increase in contribution from pair production interactions. The differences between the samples were very small in regard to their similar dominance and composition of silica and aluminosilicate minerals. However, the samples MB4 and MB6 had the highest Neff values (4.31 × 10^23^), while MB2 and MB7 had the lowest Neff values. The results show that samples with higher densities of electrons should provide a greater accumulation of interaction centers for incident photons, which can therefore result in improved attenuation of radiation and effectiveness of shielding performance.

**Figure 14:**
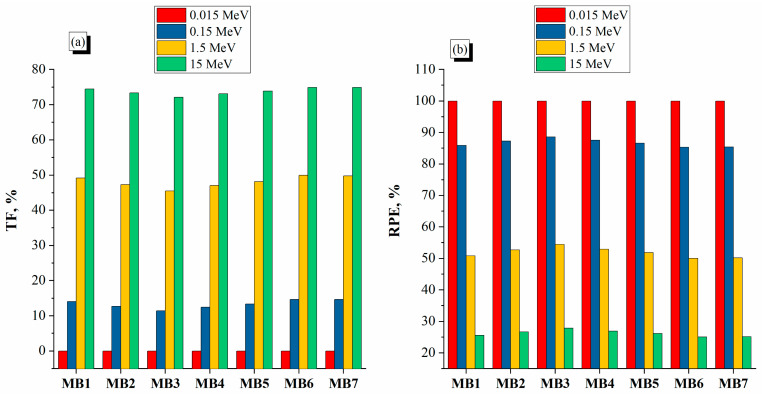
The variation of (**a**) transmission factor values (TF, %) of studied monzogranite samples at various gamma photon levels, 0.015 MeV, 0.15 MeV, 1.5 MeV and 15 MeV. (**b**) Radiation protection efficiency (RPE, %) of studied monzogranite samples at various gamma photon levels, 0.015 MeV, 0.15 MeV, 1.5 MeV and 15 MeV.

Figure 14a presents the TF values of the monzogranite samples, which were observed to have a significant relationship with the energy of the photons, with all of the samples being nearly completely attenuated at a low photon energy due to the predominance of photoelectric effects. At a low photon energy of 0.015 MeV, all monzogranite samples had TF values between 2.63 × 10^−52^% and 1.11 × 10^−45^%, indicating that almost complete attenuation occurred upon the incident photons hitting the samples. As the photon energy was increased to 0.15 MeV, the TF values of the samples ranged between 11.42% and 14.66%, indicating a decrease in the interaction probability, as well as a corresponding increase in the contribution of Compton scattering. At a photon energy of 1.5 MeV, the TF values reached 45.52% to 49.95%, and, at 15 MeV, reached 72.16% to 74.91%, therefore indicating a significant penetration of the high-energy photons through the 5 cm of monzogranite material. Among all of the samples, MB3 had the least amount of attenuation of all three photon energy levels (11.42% at 0.15 MeV; 45.52% at 1.5 MeV; and 72.16% at 15 MeV) and displays the best attenuation properties at the various energy levels. The samples MB6 and MB7, on the other hand, produced the highest TF values (approximately 74.91% at 15 MeV) of the samples, and displayed a comparatively poor ability to attenuate the various photon energy levels. The superior performance of the MB3 sample is likely due to a greater overall density and a more favorable elemental composition, which gives rise to a greater probability of interaction of the photons with the material. Lastly, the results of the TFs clearly show that the monzogranite samples are less efficient at attenuating X-ray and gamma-ray photons as photon energy increases. The sample MB3 has clearly shown the best ability of the samples analyzed to attenuate gamma-ray photons.

Figure 14b illustrates that the reverse trend expressed between TF and RPE values is a function of greater photon energy resulting in reduced probability of interacting with a material on the part of photon energy. For instance, when using photon energy of 0.015 MeV, there are no opportunities for monzogranite samples to pass through the material (virtually 100% attenuation). This illustrates that at lower energies, photoelectric absorption is more dominant than the presence of more energetic photons. For photon energy of 0.15 MeV, RPE values were in the range of 85.34–88.58%, which indicates that between the low and intermediate energy regions, excellent shielding performance was demonstrated. As photon energies increased to 1.5 and 15 MeV, RPE values dropped to between 50.05 and 54.48% and 25.09 and 27.84%, respectively, indicating that higher photon energies were less efficiently shielded by high-energy processes due to the predominance of Compton scatter and pair production processes, allowing for more successive interaction prior to escape from the material. Of all samples tested, sample MB3 provided the most efficient RPE values across all three photon energy levels (0.15, 1.5, and 15 MeV, respectively) reporting RPE values of 88.58, 54.48, and 27.84%, which demonstrates its industry leading efficacy in providing shielding from gamma radiation. On the contrary, MB6 and MB7 had the lowest RPE values throughout the evaluation (approximately 25.09% for the 15 MeV test). In addition, MB3’s favorable RPE was correlated to its density (higher), mean free path (lower), half value layer (lower), and attenuation coefficient (higher) relative to MB6 and MB7 samples. Thus, the outcome of the RPE evaluation supports the assertion that MB3 is the best monzogranite for use as gamma-ray shielding and MB7 provided lesser protective capabilities.

## 5. Conclusions

This study assesses monzogranite with respect to petrographic characterization, physical–mechanical properties and gamma-ray-shielding capability. The results of this work show that the monzogranite samples have a positive structural and radiological features. Therefore, they are good candidates for multi-functional usage in engineering. Petrographic studies reveal that all of the investigated samples are representative of typical monzogranite, and mainly composed of quartz, K-feldspar, plagioclase, biotite and hornblende. The accessory minerals in these specimens are zircon, allanite, titanite, apatite and opaque minerals. The physical properties of the examined samples exhibited relatively high densities (2.70–3.06 g/cm^3^) with low porosity (19–23%) and only minimal amounts of water absorption (12–15%). Monzogranite is highly durable and stable under significant structural loads, according to findings from mechanical tests, which showed their high strength and rigidity. Results showed that the monzogranite exhibited superior compressive strength, tensile strength, flexural strength, and elasticity due to the density of the crystalline structure, how well stitched together quartz and feldspar grains are, and how few microstructural defects are present. Collectively, these factors contribute to the resistance to deformation and failure of monzogranite. The results of the experiments indicate that the criteria of radiation protection were based only upon the interaction of photons and the introduced variable of photon energy. As such, the linear and mass attenuation coefficients decreased with increasing energy, but the half value layer (HVL) and mean free path (MFP) increased as there were less likely to be photons that will interact with the samples at higher energies. The samples that showed the most consistent superior results from a radiation protection perspective were MB3 and MB4 due to their higher densities and the fact they had considerably more Fe_2_O_3_, CaO, and K_2_O than the other samples, resulting in a greater effective atomic number and electron density of the samples. All of the physical parameters of MB3 indicated that MB3 would provide the greatest overall efficiency, as it demonstrated the highest attenuation coefficients, lowest HVL and MFP values, lowest transmission factor and highest radiation protection efficiency for the photon energy range investigated. Additionally, MAC values measured experimentally were in good agreement with the theoretical values calculated, thus validating the methodology used to make the measurements and the reliability of the Phy-X/PSD computational method used to compare the experimental and theoretical MAC values. The fact that this research has shown that monzogranite offers a combination of high strength with good gamma-ray shielding ability led to the conclusion that as a natural rock, it is a valid material for use in advanced construction applications where there is a requirement for structural and radiation protection. This study is designed to contribute to the larger body of knowledge regarding the use of sustainable radiation-shielding materials by developing an established scientific basis to determine how to choose and optimize natural granite rocks that meet the requirements of nuclear and industrial shielding.

## 6. Limitations of the Study

Although this study provided an extensive characterization of the physical, mechanical and gamma radiation-shielding properties of monzogranite by means of petrographic analyses, there remain a number of limitations in this study that need to be considered when interpreting the results presented herein. First, the testing was done under laboratory conditions; and second, no long-term assessments were performed to evaluate the durability of the material when exposed to the environment, including leaching behavior, chemical resistance to acidic and alkaline environments, thermal conductivity, coefficient of thermal expansion and thermal cycling performance. In addition, only the evaluation of gamma radiation shielding was conducted. Future evaluations should include these factors to allow for a more comprehensive understanding of the long-term performance of monzogranite in applications within nuclear, industrial, and/or other engineering environments that may be considered demanding.

## Figures and Tables

**Table 1:** Chemical analyses of major oxides (wt%) for the studied monzogranite from El-Bakriya ring complex, CED, Egypt.

S. No.	MB1	MB2	MB3	MB4	MB5	MB6	MB7
SiO_2_	71.96	72.76	73.06	72.85	71.13	73.1	72.22
TiO_2_	0.24	0.15	0.12	0.17	0.23	0.11	0.19
Al_2_O_3_	15.17	15.07	15.28	14.98	15.62	14.76	15.23
Fe_2_O_3_	2.02	1.68	1.81	1.76	2.25	1.74	1.91
MnO	0.1	0.07	0.03	0.06	0.06	0.03	0.09
MgO	0.52	0.46	0.47	0.5	0.55	0.48	0.5
CaO	1.71	1.55	1.23	1.47	1.89	1.25	1.72
Na_2_O	3.85	3.42	3.1	3.38	3.73	3.38	3.78
K_2_O	3.59	3.5	3.74	3.55	3.55	3.76	3.65
P_2_O_5_	0.13	0.11	0.11	0.11	0.14	0.1	0.12
LOI	0.68	0.56	1.1	0.72	0.94	1.03	0.81
Total	99.97	99.33	100.05	99.55	100.09	99.74	100.22

**Table 2:** Modal analysis of monzogranite from El-Bakriya ring complex, CED, Egypt.

Sample	Quartz (Q)	Alkali Feldspar (A)	Plagioclase (P)	Biotite	Hornblende	Accessories	Total	Q%	A%	P%
MB1	31	34	27	5.1	1.8	1.1	100	33.7	37.0	29.3
MB2	29	36	26	5.1	2.7	1.2	100	31.9	39.6	28.5
MB3	33	32	26	5.6	2.1	1.3	100	36.3	35.2	28.5
MB4	28	38	25	4.9	2.8	1.3	100	30.8	41.8	27.4
MB5	35	31	25	5	2.6	1.4	100	38.5	34.1	27.4
MB6	30	35	27	4.6	2.3	1.1	100	32.6	38.0	29.4
MB7	32	33	27	5.1	1.7	1.2	100	34.8	35.9	29.3
MB8	27	39	24	5.8	2.9	1.3	100	30.0	43.3	26.7
MB9	34	31	26	5.2	2.6	1.2	100	37.4	34.1	28.5
MB10	29	37	25	4.9	2.8	1.3	100	31.9	40.7	27.4
MB11	31	35	26	4.5	2.2	1.3	100	33.7	38.0	28.3

**Table 3:** Experimentally determined mechanical properties of the monzogranite samples investigated.

Sample	UCS (MPa)	Young’s Modulus, E (GPa)	Poisson’s Ratio, ν	Brazilian Tensile Strength, BTS (MPa)	Flexural Strength, MOR (MPa)	Shear Modulus, G (GPa)	Bulk Modulus, K (GPa)	Longitudinal Modulus, L (GPa)	Modulus Ratio, MR
MB1	98.32	43.5 ± 1.2	0.22 ± 0.01	8.3 ± 0.4	10.2 ± 0.5	17.8	25.9	67.2	442
MB2	200.89	58.7 ± 1.5	0.24 ± 0.01	12.6 ± 0.6	14.8 ± 0.7	23.7	37.6	90.3	292
MB3	177.96	55.4 ± 1.3	0.23 ± 0.01	11.7 ± 0.5	13.9 ± 0.6	22.5	34.2	85.4	311
MB4	180.09	56.1 ± 1.4	0.24 ± 0.01	11.9 ± 0.5	14.1 ± 0.6	22.6	36	86.2	311
MB5	224.86	63.8 ± 1.7	0.25 ± 0.01	14.1 ± 0.7	15.7 ± 0.8	25.5	42.5	97.8	284
MB6	89.28	40.6 ± 1.1	0.21 ± 0.01	7.4 ± 0.3	9.3 ± 0.4	16.8	23.5	63.0	455
MB7	240.2	66.5 ± 1.8	0.25 ± 0.01	15.2 ± 0.8	16.4 ± 0.8	26.6	44.3	102.0	277

**Table 4:** Theoretical and experimental values of mass attenuation coefficient (MAC, cm^2^/g) of the monzogranite samples at gamma photon energy 0.662 MeV, 1.173 MeV and 1.332 MeV.

Sample	Density	MAC, cm^2^/g								
		0.662 MeV			1.173 MeV			1.332 MeV		
		Theo	Exp	Diff, %	Theo	Exp	Diff, %	Theo	Exp	Diff, %
MB1	2.76	0.0767	0.0736	4.04	0.0583	0.0559	4.12	0.0546	0.0516	5.49
MB2	2.91	0.0767	0.0718	6.39	0.0583	0.0538	7.72	0.0547	0.0508	7.13
MB3	3.06	0.0767	0.0704	8.21	0.0583	0.0543	6.86	0.0547	0.0525	4.02
MB4	2.93	0.0767	0.074	3.52	0.0583	0.0562	3.60	0.0546	0.0502	8.06
MB5	2.84	0.0767	0.0727	5.22	0.0583	0.0548	6.00	0.0547	0.052	4.94
MB6	2.70	0.0767	0.0709	7.56	0.0583	0.0534	8.40	0.0546	0.0509	6.78
MB7	2.71	0.0767	0.0722	5.87	0.0583	0.0553	5.15	0.0547	0.0528	3.47

## Data Availability

The original contributions presented in the study are included in the article, further inquiries can be directed to the corresponding author.
